# How the Duration and Mode of Photopolymerization Affect the Mechanical Properties of a Dental Composite Resin

**DOI:** 10.3390/ma16010113

**Published:** 2022-12-22

**Authors:** Leszek Szalewski, Dorota Wójcik, Weronika Sofińska-Chmiel, Marcin Kuśmierz, Ingrid Różyło-Kalinowska

**Affiliations:** 1 Digital Dentistry Lab., Department of Dental and Maxillofacial Radiodiagnostics, Medical University of Lublin, 20-093 Lublin, Poland; 2 Department of Dental Prosthetics, Medical University of Lublin, 20-093 Lublin, Poland; 3 Analytical Laboratory, Institute of Chemical Sciences, Faculty of Chemistry, Maria Curie Skłodowska University, Maria Curie Skłodowska Sq. 3, 20-031 Lublin, Poland; 4 Department of Dental and Maxillofacial Radiodiagnostics with Digital Dentistry Lab., Medical University of Lublin, 20-093 Lublin, Poland

**Keywords:** restorative dentistry, aesthetic dentistry, biomechanics, composites, photopolymerization, flexural strength

## Abstract

Composite materials are the most common materials in use in modern dentistry. Over the years, the methods of photopolymerization of composite materials have been improved with the use of various devices, such as quartz tungsten halogen lamps (QTHs), light-emitting diode units (LEDs), plasma-arc lamps and argon-ion lasers. This study aimed to compare the mechanical properties of a composite material, depending on the time and mode of photopolymerization. One hundred and forty rectangular specimens (25 × 2 × 2 mm) and forty-two disc-shaped samples (5 mm diameter and 2 mm thickness) were prepared from shade A2 Boston composite resin. Samples were cured using the following seven photopolymerization protocols: four fast-cure modes (full power for 3, 5, 10, and 20 s), two pulse-cure modes (5 and 10 shots of 1 s exposures at full power), and one step-cure mode (soft start with a progressive cycle lasting 9 s). Specimens were subjected to a flexural strength test, Vickers microhardness test, and FTIR spectroscopy test. A 2-factor ANOVA and post-hoc tests were carried out to assess the differences in the flexural strength parameter between the tested groups of samples before and after aging. A mixed-model ANOVA was carried out to assess the differences in the Vickers microhardness parameter between the tested groups of samples before and after aging. The lowest values of flexural strength (*p* < 0.001) and Vickers microhardness (*p* < 0.001) were obtained for the 3 s mode for the pre- and post-aging groups. The FTIR mapping tests showed a much more homogeneous chemical structure of the composite after 20 s of continuous irradiation, compared to the sample irradiated for 5 s in the continuous mode. The mode and cure time affects the mechanical properties of the composite resin. Appropriate selection of the cure mode and time ensures better mechanical properties of composite resin. This suggests that the survival of dental restorations within the oral cavity could be extended by using longer photopolymerization durations.

## 1. Introduction

Composite materials are the most common materials in use in modern-day dentistry and were introduced in the 1960s. Since that time, their composition has been modified to achieve the best mechanical, biological, and aesthetic properties [1,2]. At present, composite materials are used not only in conservative dentistry, but also in other areas of dentistry, such as prosthetics, periodontology, and dental surgery [1,3].

Dental composite resins are mixtures of inorganic fillers, organic matrices, coupling agents, and some additives (photoinitiators, stabilizers and inhibitors). The most frequently used filler is silicon dioxide (SiO_2_), due to its matched refractive index with a resin matrix, good stability and mechanical properties, and easy production [4]. Organic matrices are composed of different monomers, such as BisGMA, UDMA, TEGDMA, and DMAEMA [1].

Since the tensile strength of the composites is much lower than their compressive strength and typically much more sensitive to internal flaws, it is probably the most appropriate property to be assessed in strength testing [5]. However, flexure testing, a potentially simpler method that is highly related to tensile failure, is usually substituted in its place. Flexure testing is the standard means for the strength testing of dental composites (ISO 4049) and has been shown to correlate with material wear in some studies [5]. Manufacturers continue upgrading their composite materials using different resins or fillers. Sideridou et al. confirmed that the higher the percentage content of filler, the higher the flexural strength [6]. The final properties of composites may be affected by the material composition, as well as by the photopolymerization mode [7,8]. The origins of light-polymerization date back to the 1970s, when UV light (with a wavelength of approx. 365 nm) was used to polymerize composite resins [7]. Over the years, the methods of photopolymerization of composite materials have been improved with the use of various devices, such as quartz tungsten halogen lamps (QTHs), light-emitting diode units (LEDs), plasma-arc lamps, and argon ion lasers (with wavelengths from 360 to 560 nm) [9]. Numerous manufacturers continued to introduce increasingly advanced lamps to the market, modifying photopolymerization programs and the power of devices to ensure the best mechanical parameters of composite materials. However, many dentists fail to use their photopolymerization lamps properly by focusing mainly on the anatomical shape and the aesthetics of fillings [9]. In a survey study by Kopperud et al., nearly one-third of the dentists did not properly protect their eyes against the blue light, while 78.3% of the responders were unaware of the irradiance values of their photopolymerization lamps [9]. Numerous responders never verified the quality of the light emitted by their photopolymerization lamps. An incorrect angle of the lamp tip during polymerization or a malfunctioning lamp can cause a significant reduction in the energy that reaches the composite material, and thus disturb the polymerization process. This may result in poorer mechanical properties of the material. Other studies showed that pre-clinical dental students and dentists in their internship year improperly use photopolymerization lamps, not delivering the required energy to the composite layer [10].

With the development of equipment, the duration of photopolymerization has also changed. For years, 20 s of irradiation was the gold standard, whereas new photopolymerization lamps offer photopolymerization times reduced to 3 s [3]. The mechanical properties of composite resins must improve or remain at the same level after technological changes. New solutions that could deteriorate the mechanical properties of materials should not be introduced. A shortened duration is not a sufficient reason to use such solutions.

This study aimed to compare the mechanical properties of a composite material depending on the duration and mode of photopolymerization before and after artificial aging. The null hypothesis was as follows: (1) the different modes and durations of photopolymerization would not affect the flexural strength, Vickers microhardness, degree of conversion, or chemical structure of the composite resin; (2) there is no difference “before and after artificial aging” in flexural strength, Vickers microhardness, degree of conversion or chemical structure of the composite resin.

## 2. Materials and Methods

**Figure 1:**
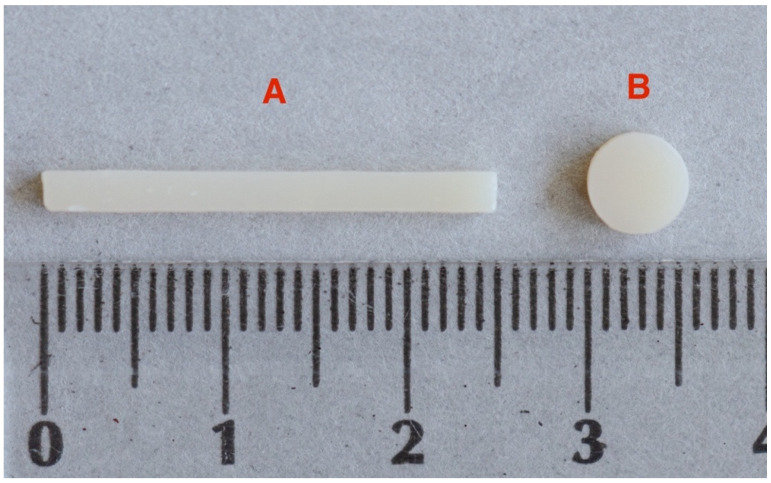
Pictures of samples: (**A**) rectangular specimen for flexural strength test; (**B**) disc-shaped specimen for Vickers microhardness test.

One hundred and forty specimens have been prepared for flexural strength tests and forty-two specimens were prepared for Vickers microhardness tests, as per the ISO 4049:2010-12 standard, from shade A2 Boston composite resin (Arkona LFS, Nasutów, Poland). The resin is a nano-hybrid composite with a 78% filler content (barium–aluminium–silicon glass, fumed silica; titanium dioxide) with a particle size of 15–2000 nm. The matrix comprises Bis-GMA, UDMA, Bis-EMA, and TEGDMA resins. Rectangular specimens (25 × 2 × 2 mm) and disc-shaped samples (5 mm diameter and 2 mm thickness) were produced using a steel mold placed on a microscope slide to achieve a flat surface (Figure 1). Subsequently, one portion of composite resin was condensed with a dental plugger and flattened by being pressed by the slide. The composite material was then polymerized through a layer of polyethylene film (40 μm) to eliminate oxygen inhibition on the surface. Samples were polymerized using a high-powered LED LCU (Mini LED III Supercharged, Acteon Group, Merignac, France). Samples were cured using the following 7 photopolymerization protocols: 4 fast-cure modes (full power for 3, 5, 10, and 20 s), 2 pulse-cure modes (5 and 10 shots of 1 s exposures at full power), and 1 step-cure mode (soft start with a progressive cycle lasting 9 s) (Table 1). A total of 20 rectangular and 6 disc-shaped samples were made for each mode and time of photopolymerization. According to the manufacturer’s information, the photopolymerization power was 2000 mW/cm^2^ when a 7.5 mm diameter tip was used. Each rectangular sample was polymerized at 4 points (in a longitudinal direction every 6 mm), and the disc-shaped specimens were polymerized at 1 point.

**Figure 2:**
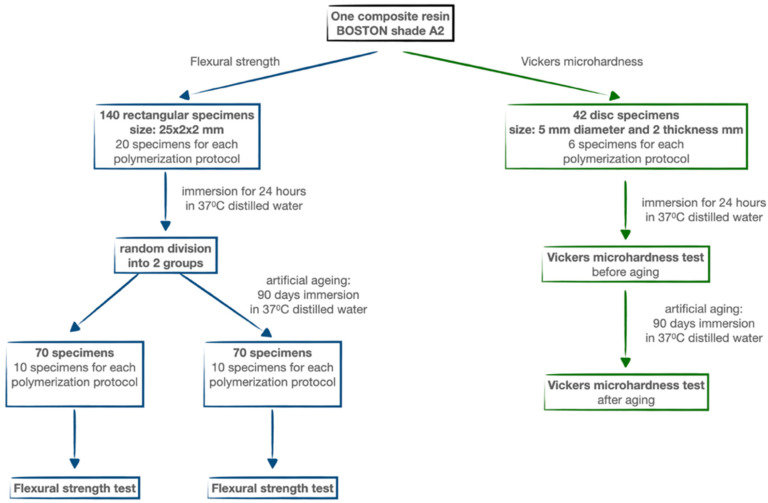
Flow chart of the research process.

After photopolymerization, the specimens were released from the mold. The steel mold blocked the light from the side of the samples, which made it possible to obtain reproducible results. There was no risk of false results due to lateral polymerization of the sample. Next, the specimens were examined for the presence of air bubbles and defective specimens were excluded from the study. The specimens were immersed in distilled water at the temperature of 37 °C for 24 h. One-half of the rectangular specimens were immediately subjected to flexural strength tests, while the rest of the specimens were artificially aged. Artificial aging included the immersion of the specimens for 90 days in distilled water at 37 °C. After this time, the specimens were dried and tested for flexural strength. All disc-shaped specimens were subjected to Vickers microhardness testing, and subsequently immersed in distilled water at 37 °C for 90 days. After this time, specimens were once again tested for Vickers microhardness (Figure 2).

### 2.1. Flexural Strength Test

Flexural strength was tested by three-point bending tests using the Cometech QC-508M2 (Cometech Testing Machine, Taichung, Taiwan) testing machine with an opening width of 20 mm, initial gripping force of 1 N, and a crosshead speed of 0.75 mm/min. Each sample was measured before the test. Only samples with dimensions that met the criteria of 2 mm ± 0.01 mm were tested (6 specimens were rejected). The exact measurements of each specimen were entered into the equation to calculate the flexural strength value. The test end was marked by the specimen being crushed. Flexural strength was calculated using the following equation:

S = 3Fl/2bh^2^

where F is the maximum load exerted on the specimens in Newton;

l is the distance (20 mm) between the supports, with an accuracy of ±0.01 mm;

b is the width (2 mm ± 0.01 mm) of the specimens measured before testing;

h is the height (2 mm ± 0.01 mm) of the specimens measured before testing.

### 2.2. Vickers Microhardness Test

The microhardness of the tested samples was evaluated on the Vickers scale using a Pruftechnik KB-10 durometer (KB Pruftechnik GmbH, Hochdorf-Assenheim, Germany) with a load force of 200 g (1.962 N), which operated for 15 s. In cases of an uneven or illegible probe footprint, the measurement was repeated. The diagonal length was measured using the light microscope Matrix Vision 5MP (Matrix Vision GmbH, Oppenweiler, Germany).

### 2.3. Fourier Transform Infrared (FTIR) Spectroscopy

To observe the process of photopolymerization of dental resin composites as a result of irradiation with a high-powered LED LCU photopolymerization lamp (Mini LED III supercharged, Acteon Group, Merignac, France), Fourier transform infrared spectroscopy–attenuated total reflectance (FTIR–ATR) spectroscopic tests were carried out. The prepared composite samples (disc-shaped, 5 × 2 mm) with the following durations and modes were tested using the FTIR Thermo Nicolet 8700 (Thermo Scientific, Waltham, MA, USA) spectrometer with the Smart Orbit™ attachment diamond ATR and a DTGS (deuterated triglycine sulfate) detector: uncured, 3, 5, 10 and 20 s continuous mode, 5 and 10 s pulsed mode, and 9 s slow-start mode. The tests were carried out directly from the surface of the composites at room temperature. FTIR spectra were obtained using the ATR technique with a diamond crystal in the wavenumber range of 4000–400 cm^−1^, with a spectral resolution of 4 cm^−1^. A total of 64 scans were obtained for each spectrum. The obtained spectra were subjected to ATR correction, automatic baseline correction, and normalization using the Omnic SpectaTM software 833-036200.

### 2.4. FTIR Microscopy

Chemical maps of the composites were made for the samples irradiated for 3 and 20 s in the continuous mode. The tests were carried out using the FTIR iN10 MX microscope (Thermo Scientific) with the specular reflection method at room temperature, using the MCT-A detector cooled with liquid nitrogen. A total of 16 scans were obtained for each spectrum. For the sample irradiated for 3 s in the continuous mode, the spectra were collected from the area of 10,000 × 9000 µm. The map was created using the correlation method with the peak at the position 2956 cm^−1^. For the composite sample irradiated for 20 s in the continuous mode, the spectra were collected from the area of 10,000 × 7000 µm. The map was created using the correlation method with the peak at the position 2951 cm^−1^.

### 2.5. XPS Spectroscopy

X-ray photoelectron spectroscopy (XPS) studies were performed using a multi-chamber UHV system (PREVAC, Rogów, Poland). Spectra were collected using a hemispherical Scienta R4000 electron analyzer (Scienta Omicron, Uppsala, Sweden). A Scienta SAX-100 X-ray source (Al Kα, 1486.6 eV; 0.8 eV band) equipped with an XM 650 X-ray Monochromator (PREVAC, Rogów, Poland) (0.2 eV band) was used as complementary equipment.

The pass energy of the analyzer was set to 200 eV for survey spectra (with a 500 meV step), and 50 eV for the C1s region (high-resolution spectra with a 50 meV step). The base pressure in the analysis chamber was 5·10^−9^ mbar. During the spectra collection, it was not higher than 3·10^−8^ mbar.

### 2.6. Statistical Methods

To assess flexural strength, a comparative analysis of the mean values of flexural strength (MPa) was carried out. The groups were compared regarding two different factors, namely the mode of photopolymerization (7 different modes) and the aging process (before and after). A 2-factor ANOVA and post-hoc tests (Bonferroni test) were performed. The use of 2-ANOVA is acceptable due to the fulfillment of the normality condition and very high partial eta2 values. We conducted a post-hoc power analysis and for the effects of aging in this analysis, due to the compared parameters and interactions, the obtained power (1 − β) ranged from 0.87 to 0.99. The effect of interactions between individual factors was also considered. Due to the small number of observations, great care was when interpreting the discovered associations. The hypotheses were assessed using the significance level of *p* = 0.05. Mean values and standard deviations were calculated. The data were analyzed for normal distribution (Shapiro–Wilk’s test) with the determination of skewness and kurtosis parameters. In addition, the homogeneity of variance was also investigated.

In the analysis of the Vickers microhardness test results, hypotheses related to the differences between the mean values obtained for individual photopolymerization modes and the aging effect were verified by mixed-model ANOVA assumptions analysis, followed by mixed-model ANOVA and post-hoc tests. The hypotheses were verified using the significance level of *p* = 0.05. Mean values and standard deviations were calculated. In the first step, the normality of the distribution was analyzed using the Shapiro–Wilk’s test, along with the analysis of skewness and kurtosis. Skewness and kurtosis are two parameters that should be considered for samples with a small number of observations. In addition, Levene’s test was included in the analyses to assess the homogeneity of the variance. The analyses were conducted using IBM SPSS Statistics 27 software (International Business Machines Corporation, New York, NY, USA).

## 3. Results

The measurements identified as outliers were removed. For pre-aging observations, a total of three cases were removed from the results pool, including one for the 3 s fast-cure mode, 5 s fast-cure mode, and 5 s pulse-cure mode. The results of the normality test did not allow us to reject the null hypothesis about the normality of the distribution. For the post-aging measurement, one finding was identified as an outlier for the 3 s fast-cure mode. For the post-aging measurement, the distribution normality test results were statistically significant at *p* < 0.05 for the 5 s fast-cure and 10 s fast-cure modes, although the significance was not lower than *p* = 0.01. Taking this into account, and as part of exercising caution when interpreting the obtained results, a 2-factor ANOVA was performed.

The results for the flexural strength variable were indicative of statistically significant differences due to the aging process (F (1, 122) = 660.4; *p* < 0.001; η^2^ = 0.84). Greater strengths were measured in the pre-aging tests. In addition, the photopolymerization modes were also indicative of the significant difference in the strength level (F (6, 122) = 7.47, *p* < 0.001; η^2^ = 0.27). A post-hoc analysis using the Bonferroni test was performed to compare the flexural strengths for different photopolymerization modes. The lowest flexural strength value was measured for the 3 s fast-cure mode (M = 84.17; SD = 3.18). The flexural strength measured for this mode was significantly different from those measured for other modes, except for the 5 s fast-cure mode (M = 94.62; SD = 3.1). The highest value of flexural strength was measured for the 20 s fast-cure mode (M = 110.2; SD = 3.01). However, the difference was statistically significant only when compared to the 3 s fast-cure and 5 s fast-cure modes. Other differences in the comparisons between individual modes were found not to be statistically significant.

**Figure 3:**
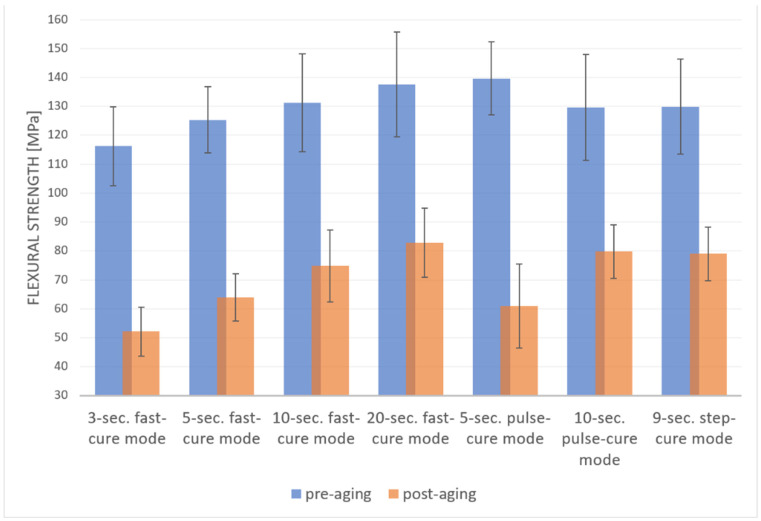
Mean flexural strength values (MPa) and standard deviation (error bars) (MPa) for photopolymerization mode and pre-/post-aging status.

The interaction effect (aging × photopolymerization mode) was observed to be of statistical significance (F (6, 122) = 2.62, *p* = 0.02; η^2^ = 0.11). The differences were calculated using the post-hoc Bonferroni test. Significant differences in flexural strengths were observed for each of the seven photopolymerization modes (Figure 3).

**Figure 4:**
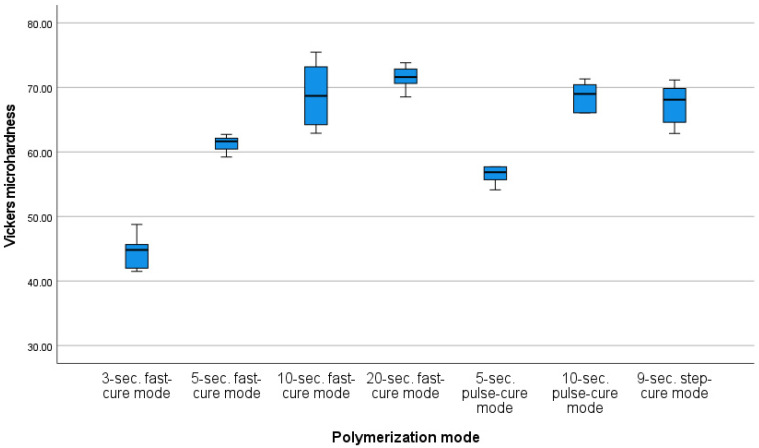
Mean Vickers microhardness values for different photopolymerization modes (pre-aging).

**Figure 5:**
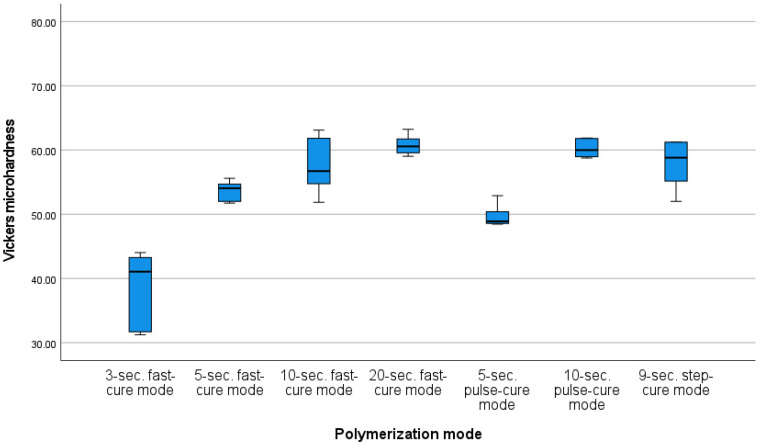
Mean Vickers microhardness values for different photopolymerization modes (post-aging).

**Figure 6:**
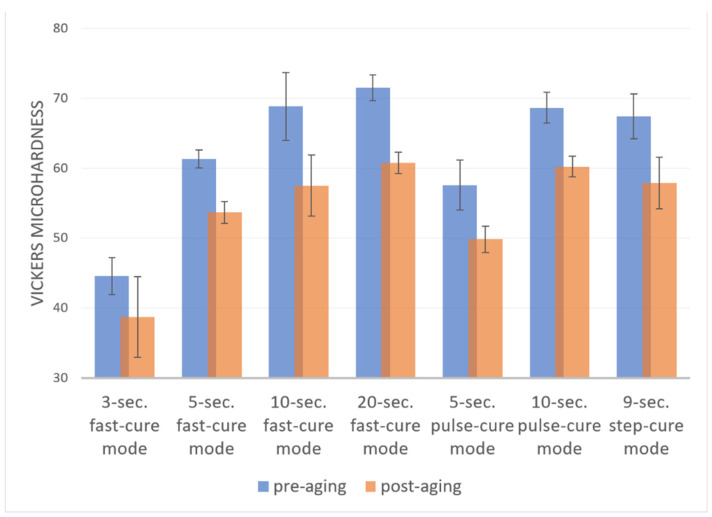
Change in mean Vickers microhardness values as the result of aging in groups that corresponded to different photopolymerization modes (*p* < 0.001).

Before calculating the Vickers microhardness test results, an analysis of the measurements in terms of the occurrence of outliers, normality of distribution and skewness was performed to characterize the samples under individual conditions. One finding identified as an outlier was removed from the pool. In each group, the distribution of results did not deviate from the normal distribution and the values of skewness and kurtosis were considered to be acceptable. A mixed-model analysis of variance was carried out to verify the hypothesis that the photopolymerization method has an impact on the Vickers microhardness measurements. The result of Levene’s test suggested that the assumption of the homogeneity of variance was not met for the post-aging mean values. The results were statistically significant for both the pre-aging and post-aging photopolymerization processes (F (6, 524.75) = 58.27; *p* < 0.001; η^2^ = 0.82; and FWelch (6, 14.6) = 30.54; *p* < 0.001; η^2^ = 0.85, respectively). There was also a significant change in the Vickers microhardness parameters as a result of aging (λ = 0.86, F (1, 34) = 215.27; *p* < 0.001; η^2^ = 0.86). However, the effect of the interaction (ageing × polymerization mode) was statistically insignificant (*p* > 0.05), indicating that the pattern of differences before aging corresponds to that after aging. The differences in the mean pre-aging Vickers microhardness values are shown in Figure 4. The differences in the mean post-aging Vickers microhardness values are shown in Figure 5. The change in mean values as the result of the aging process is presented in Figure 6.

The lowest Vickers microhardness value was measured for the 3 s fast-cure mode (M = 44.59, SD = 2.65); it was significantly lower than the values measured for all the other modes. The second-lowest Vickers microhardness value was obtained for the 5 s pulse-cure mode (M = 57.58, SD = 3.57). In this case, the Vickers microhardness value was statistically significantly different from those obtained for the other modes, except for the 5 s fast-cure mode. The Vickers microhardness value measured for the 5 s the fast-cure mode was also significantly different (lower) than those obtained for the other photopolymerization modes. In contrast, no statistically significant differences in Vickers microhardness values were observed for the 10 s fast-cure mode, 20 s fast-cure mode, 10 s pulse-cure mode or 9 s step-cure mode.

The analysis of differences in Vickers microhardness values resulting from the use of different modes of post-aging photopolymerization was carried out using a post-hoc test with the Games–Howell correction. The correction was applied due to the breach of the homogeneity of variance.

In addition, in this case, the lowest Vickers microhardness value was measured for the 3 s fast-cure mode (M = 38.73, SD = 5.8); it was significantly lower than the values measured for all the other modes. The second-lowest Vickers microhardness value was obtained for the 5 s pulse-cure mode (M = 49.85, SD = 1.88). Significant differences were observed between this mode and the 3 s fast-cure mode, 20 s fast-cure mode, 10 s pulse-cure mode and 9 s step-cure mode. However, no difference was observed between the average Vickers microhardness value obtained by this photopolymerization mode and the 5 s fast-cure mode, or the 10 s fast-cure mode. The 5 s fast-cure mode (M = 53.69, SD = 1.56) differed significantly from the 3 s fast-cure mode, 20 s fast-cure mode, and 10 s pulse-cure mode. On the other hand, the 10 s fast-cure mode (M = 57.5, SD = 4.39) only differed significantly from the 3 s fast-cure mode. The highest Vickers microhardness values were obtained for the 10 s pulse-cure mode (M = 60.23, SD = 1.48) and the 20 s fast-cure mode (M = 60.78, SD = 1.52).

**Figure 7:**
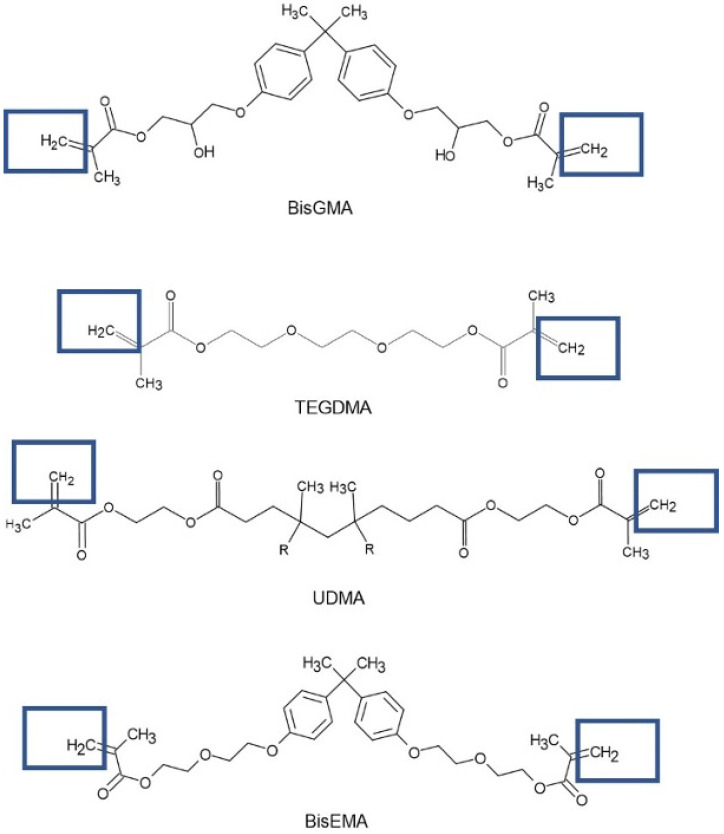
Chemical formulae of the resins included in the composites with the denoted bonds involved in the photopolymerization process.

The chemical formulae of the resins BisGMA, TEGDMA, UDMA, and BisEMA, which were the main materials that underwent the photopolymerization process as a result of irradiation, are shown in Figure 7. The figure shows the chemical bonds involved in the photopolymerization process.

**Figure 8:**
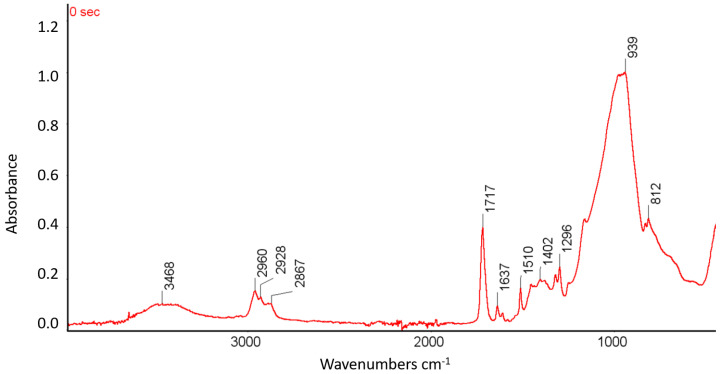
FTIR-ATR spectrum of the composite before irradiation with the denoted spectral bands.

To observe the photopolymerization process that occurs as a result of different modes of irradiation of dental composite resin, FTIR spectroscopic tests were carried out. Figure 8 presents the FTIR spectrum obtained using the ATR technique for the composite before the irradiation process.

**Figure 9:**
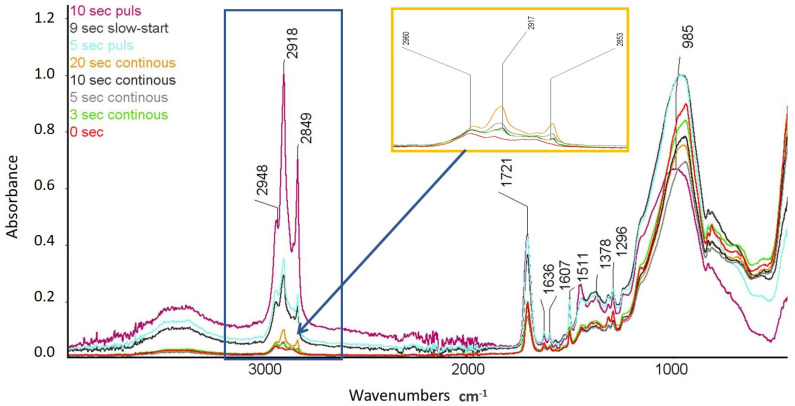
Comparison of the FTIR-ATR spectra of composites with the denoted bands involved in the photopolymerization process.

The FTIR studies proved the presence of signals that were characteristic of the chemical bonds present in the resins and the composite filler. Bands that were characteristic of the stretching vibrations of OH groups in the wavenumber range of 3700–3200 cm^−1^ and those characteristics of the stretching vibrations of -CH_3_ and -CH_2_ groups in the range of 3000–2700 cm^−1^ were observed. An intense peak that corresponded to the C=O stretching vibrations in the range of 1900–1600 cm^−1^ was found. The FTIR spectra also showed the presence of C=C stretching vibrations in the wavenumber range of 1690–1580 and the presence of aromatic ring vibrations in the wavenumber range of 1550–1450 cm^−1^. The tests also exhibited the presence of signals that were characteristic of the CH_3_ groups in the range of wave numbers 1470–1350 cm^−1^. Signals that were characteristic of silicon present in the composite filler were also observed in the form of an intense peak in the wavenumber range of 1100–900 cm^−1^. Figure 9 presents the summary of FTIR-ATR spectra before and after irradiation with the denoted bands involved in the photopolymerization process.

The FTIR-ATR tests showed an evident increase in the intensity of the peak that corresponded to the stretching vibrations of the -CH_2_ groups after irradiation of the composites, which indicates the photopolymerization process. As a result of the photopolymerization process of the composite, the quantity of C=C double bonds decreases, and the quantity of -CH_2_ bonds increases. The lowest peak intensity at the position of 2948 cm^−1^ was observed for the unexposed composite. The highest peak intensity was found for the composite irradiated for 10 s in the pulsed mode. With the increase in the irradiation duration of the composite in the continuous mode, there was an increase in the intensity of the peak at the position of 2948 cm^−1^. The tests proved the much higher degree of photopolymerization of the composite using the pulsed and slow-start mode compared to the continuous mode.

**Figure 10:**
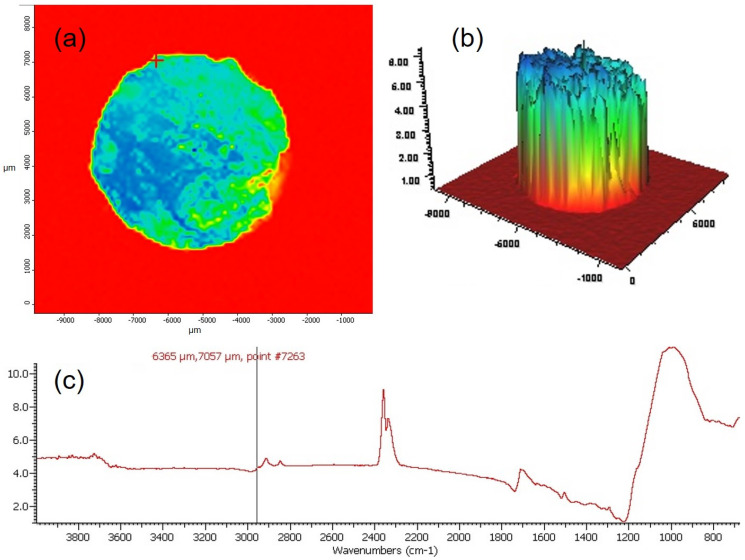
Results of FTIR mapping tests, in which the composite samples were exposed to the continuous mode for 5 s: (**a**) chemical map obtained using the correlation method based on the peak at the position of 2960 cm^−1^, (**b**) 3D mapping image; (**c**) FTIR spectrum obtained using the specular reflection technique.

**Figure 11:**
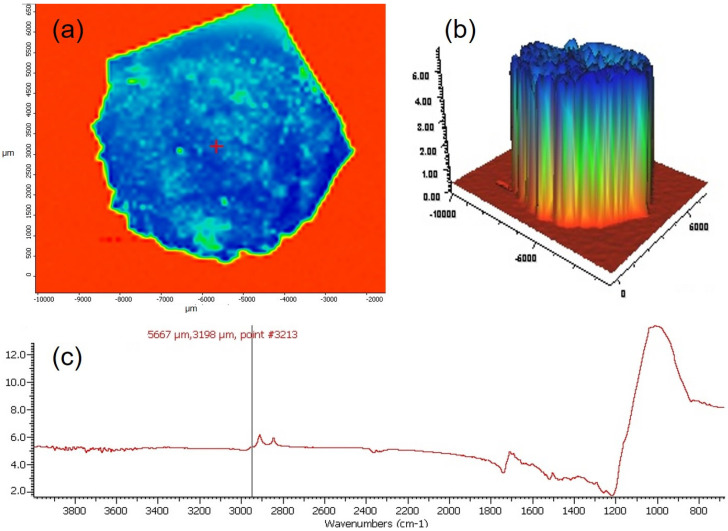
Results of FTIR mapping tests, in which the composite samples were exposed to the continuous mode for 20 s: (**a**) chemical map obtained using the correlation method based on the peak at the position of 2960 cm^−1^, (**b**) 3D mapping image; (**c**) FTIR spectrum obtained using the specular reflection technique.

To observe the photopolymerization process on the entire surface of the composite, tests were carried out using the FTIR Nicolet In10MX microscope. The tests referred to the sample irradiated for 5 s in the continuous mode and 20 s in the continuous mode. The test results are shown in Figure 10 and Figure 11.

The tests showed a much more homogeneous chemical structure of the composite after 20 s of continuous irradiation compared to the sample irradiated for 5 s in the continuous mode. These differences are visible on both chemical maps and 3D profiles of the tested samples. Smaller differences in the signal intensity were observed for the sample irradiated for 20 s in the continuous mode compared to that irradiated for 5 s in the continuous mode.

### XPS Spectroscopy

**Figure 12:**
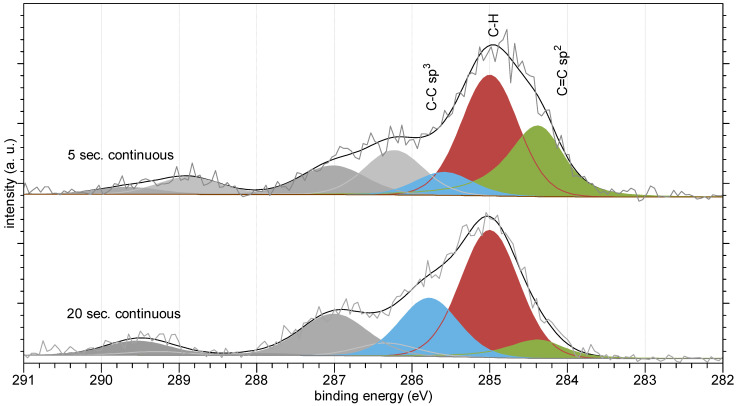
High-resolution XPS spectra of C1s regions: sample “5 s continuous” and “20 s continuous”.

As stated before, the hardening of a resin involves terminal carbon atoms. Their double bonds break, and single C-C bonds are created. Simultaneously, the hybridization of these carbon atoms changes from sp^2^ to sp^3^. “Fresh” and “hardened” resin states can be detected using XPS spectroscopy because the two carbon states are distinguishable by this method. Figure 12 presents high-resolution spectra of the following two selected samples: “5 s continuous mode” and “20 s continuous mode”.

A set of peaks used to recreate spectra envelopes was based on publications by Koinuma [11], Briggs [12], and Beamson [13]. The following three peaks are of special interest: C-C sp^3^ (blue), which corresponds to carbon atoms building the main resins’ chains, C-H (red), which corresponds to hydrogenated terminal carbon atoms, and C=C sp^2^, which corresponds to terminal, double-bonded carbon atoms.

Figure 12 shows that the C=C sp^2^ peak is significantly larger in the case of the less irradiated, thus less hardened, first sample (i.e., “5 s continuous”), clearly indicating that these carbon atoms are more numerous compared to the more hardened, second sample (20 s continuous). Simultaneously, the C-C peak is larger in the case of the second sample; the number of C-C sp^3^ atoms is greater because they are the product of hardening.

## 4. Discussion

The experimental results show that the change in the photopolymerization time has a great impact on the mechanical properties of the resin, such as flexural strength and Vickers microhardness. Hence, the null hypothesis was rejected. Probably, the total amount of energy generated during the photopolymerization of the composite materials is significant. The comparison of different photopolymerization modes with similar photopolymerization times that translate to similar total energies reveals (5 s fast-cure and pulse-cure modes—10 J/cm^2^ and 10 s fast-cure and pulse-cure modes—20 J/cm^2^) no statistically significant differences in the mechanical parameters of the composite materials. Bauer et al. demonstrated that the photopolymerization of composite materials should be no shorter than 20 s to achieve values of flexural strength equal to the ISO standard [14]. In this study, the authors used a medium-power LED curing light (1261 mW/cm^2^). This was also confirmed by Bhamra et al., who showed that providing the required dose of irradiation is necessary for proper photopolymerization and that increasing this dose does not significantly improve the mechanical properties of the material [15]. So, the higher the lamp power, the shorter the required photopolymerization times. In our study, values of flexural strength higher than those required for composite materials according to ISO 4049:2010-12 (80 MPA) were obtained for all photopolymerization modes.

Dental resins polymerized with continuous modes show better mechanical properties in comparison with those obtained by pulsed and step-cure photopolymerization modes, as confirmed by Dos Santos et al. [16]. This was also confirmed by the results of our study. On the other hand, Ivanisevic et al. demonstrated that the Vickers microhardness of the surface of materials polymerized using the step-cure method is higher than that of samples polymerized using the continuous mode [17]. However, different results were obtained depending on the place of microhardness measurements. In the case of measurements performed at the bottom of the samples, higher values were observed in the group of samples that were continuously polymerized [17]. Nonetheless, the microhardness of the outer surface appears to be of the greatest importance, as this is the surface that would remain in contact with the oral cavity environment. Both studies agree that the longer the photopolymerization times, the higher the microhardness values obtained, regardless of the photopolymerization mode used [16,17].

Par et al. studied the effect of prolonged photopolymerization (30 s) on Vickers microhardness values and temperature increases [18]. They showed that the total energy delivered to the material increases with the photopolymerization time, contributing to increased microhardness and temperature values. This is in line with the results of our study, which demonstrate the effect of photopolymerization time on the microhardness values [19]. The increase in the composite temperature as a function of the photopolymerization times and modes had been confirmed by our previous studies.

According to Almeida et al., the mechanical properties of composite materials can also be improved by heating the material to 60 °C before photopolymerization [20]. This will improve both the flexural strength and the microhardness values (as measured using the Knoop scale). These results are also consistent with those reported by other authors [21,22].

The choice of the photopolymerization device has an impact not only on the mechanical properties of materials, but also, for example, on marginal leakage. As shown by Yilmaz et al., marginal leakage was minimized with the use of a 1300 mW/cm^2^ LED lamp, as compared to 500 mW/cm^2^ LEDs and quartz tungsten halogen lamps, despite the longer photopolymerization times used in the case of the latter two types of devices being twice as long [23]. The photopolymerization time can strongly influence the Vickers microhardness, which increases with the photopolymerization times when extended from 20 to 40 and 60 s, as demonstrated by Hammouda [24]. In contrast, Peutzfled et al. [25] and Cuevas-Suárez et al. [26] demonstrated that short photopolymerization times with high irradiation powers may lead to lower microhardness values, as compared to lamps with lower irradiation power but longer photopolymerization times, if similar total energies are delivered to the material in both cases.

During composite photopolymerization, not all double-carbon bonds are converted into single-carbon bonds, which is considered as the degree of conversion. The literature reports that the degree of conversion varies between 14 and 44% after the photopolymerization of composite resins [27]. In turn, 10% or more of unreacted double bonds result in the release of residual monomers [28]. The composition of the composite dental resins influences the degree of conversion and the residual monomer. In our research, all samples were made of the same material, which made it possible to standardize the test scheme, but it is not known whether we would obtain similar results using materials with different resin compositions. The resins of the matrix of composite resin are characterized by different degrees of conversion, as demonstrated by Gawriołek et al. [29]. The lowest degree conversion values 24 h after photopolymerization was obtained for Bis-GMA (34.5%) and the highest for TEGDMA (82.5%). Increasing the degree of conversion can be achieved by extending the photopolymerization time to 40 s [30], or by changing the filler to a more UV transmissive filler [31]. Our research also confirms the rule that the longer the photopolymerization time, the higher the degree of conversion. The degree of resin conversion has a significant positive effect on the mechanical properties of composite materials, such as flexural strength or water sorption [29]. According to various studies, the type of curing lamp does not affect the conversion, but the amount of energy has a significant impact on the degree of conversion, as demonstrated by Yoon et al. [32]. Our study also confirms that longer photopolymerization times, and thus higher energy doses, increase the degree of conversion. In addition, our research suggests that the photopolymerization mode may affect the degree of conversion, but this needs to be tested with different composite resins.

This study has been burdened by certain limitations due to the simplified laboratory model used. The research did not consider factors such as the residual monomer fraction, which may affect the quality of the photopolymerization of composite resins. However, Sonkaya et al. showed in their studies that the photopolymerization time did not affect the residual monomer. According to this study, only the material composition influenced the residual monomer [33]. Since we used one dental composite material in the study, we omitted the residual monomer analysis.

When assessing the aging of materials, chemical and thermal factors associated with food consumption and the effect of brushing were not considered. The pH of the drinks and meals that we consume can have a great influence on the mechanical properties of composite materials [34]. Brushing, particularly using hard brushes and abrasive whitening pastes, may also lead to changes in the microhardness of composites. According to the study by Salam et al., most of the whitening toothpastes tested contributed to the increased microhardness of composite materials; however, some of them reduced the values of this parameter in a statistically significant manner [35].

The appropriate choice of the mode and duration of photopolymerization of composite resins affects the mechanical properties of the resin. Particularly regarding the final composite filling layers, the clinicians should choose longer photopolymerization times (10; 20 s) to obtain the best mechanical properties and degree of conversion. As a result, the survival of dental restorations within the oral cavity could be extended.

## 5. Conclusions

Within the limitation of this study that examined only one dental resin, the following conclusions can be drawn:

1. Flexural strength and Vickers microhardness of the tested resin are mainly determined by the duration of photopolymerization. A shorter duration of curing led to lower values of flexural strength and Vickers microhardness.
2. The best mechanical properties of the tested resin before and after aging were obtained in the 20 s fast-cure mode.
3. The values of flexural strength and Vickers microhardness of the tested resin decreased after artificial aging, in particular in the pulse-cure 5 s mode.
4. The values of the degree of conversion of the tested resin were higher using the pulsed and slow-start mode, compared to the continuous mode photopolymerization.
5. The values of the degree of conversion of the tested resin increased with increasing duration of continuous mode photopolymerization.
6. The chemical structure of the tested resin was more homogenous with a longer duration of photopolymerization.

## Figures and Tables

**Table 1:** Characteristics of the photopolymerization modes.

Mode	Description	Total Energy (J/cm^2^)
Fast-Cure 3s	Full power for 3 s	6
Fast-Cure 5s	Full power for 5 s	10
Fast-Cure 10s	Full power for 10 s	20
Fast-Cure 20s	Full power for 20 s	40
Pulse-Cure 5s	5 shots of 1 s (full power with emission of 5/10 successive one-second flashes with a rest of period of 250 ms between each flash)	10
Pulse-Cure 10s	10 shots of 1 s (full power with emission of 5/10 successive one-second flashes with a rest of period of 250 ms between each flash)	20
Step-Cure 9s	6 s progressively and 3 s at full power	12

MINI LED III Supercharged, Acteon Group, France; wavelength range: 420–480 nm; central wavelength: 455–465 nm; intensity: 2000 mW/cm^2^ ± 10% for an active fiber diameter of 7.5 mm.

## Data Availability

Not applicable.
